# New Single-Layered Paper-Based Microfluidic Devices for the Analysis of Nitrite and Glucose Built via Deposition of Adhesive Tape

**DOI:** 10.3390/s19194082

**Published:** 2019-09-21

**Authors:** Peng Yu, Muhan Deng, Yi Yang

**Affiliations:** Key Laboratory of Low-Dimensional Materials and Application Technologies (Ministry of Education), School of Materials Science and Engineering, Xiangtan University, Xiangtan 411105, China; 201821551419@smail.xtu.edu.cn (M.D.); yangyi@xtu.edu.cn (Y.Y.)

**Keywords:** paper-based microfluidic devices, colorimetry, adhesive tape, nitrite, glucose

## Abstract

A simple, low-cost technique has been developed for the rapid fabrication of single-layered paper-based microfluidic devices (μPADs). This technique, for the first time, made use of the deposition of patterned adhesive tape into the filter paper to construct hydrophobic barriers, with the help of toluene. Unlike other reported multi-layered μPADs that merely made use of adhesive tape as a separate layer for sealing or fluid flow controlling, the patterned adhesive tape was simultaneously dissolved and penetrated into the filter paper, which resulted in the successful transfer of the pattern from the tape to the filter paper. To demonstrate the effectiveness of this approach, nitrite and glucose were individually measured; detection limits as low as 0.015 ± 0.004 mM and 0.022 ± 0.006 mM were reported for nitrite and glucose, respectively. Multiplexed analysis of both analytes was also carried out with respective detection limits of 0.048 ± 0.005 mM and 0.025 ± 0.006 mM for nitrite and glucose. The application of the method was demonstrated by measuring nitrite and glucose in spiked artificial urine samples and satisfied recovery results were obtained.

## 1. Introduction

Paper-based microfluidic analytical devices (μPADs) have been widely used in the scientific community, since Whitesides group first proposed it in 2007 [1]. μPADs have a number of merits, such as ease of use, low volume, biodegradability, portability, disposability, and good biocompatibility [2]. In recent years, a variety of analytical methods have been reported to be integrated with μPADs, including as electrochemistry [3], colorimetry [4], fluorometry [5], surface plasmon resonance (SPR) [6], chemiluminescence [7], electrophoresis [8], chromatography [9], and mass spectrometry (MS) [10]. Among these methods, colorimetry is most attractive due to its instrumental advantages (portability and capacity of operating analysis via scanner, camera, or smartphone). It relies on color changes that occur because of the reaction between sensing molecules and the analyte [11]. To realize the full capability of colorimetric devices, the color changes are digitally scanned/photographed and then analyzed while using image processing software [12]. Currently, the colorimetric μPADs are being utilized in various fields of analytical research [13,14,15,16,17].

Many different techniques have been reported to fabricate hydrophilic and hydrophobic microstructures on paper substrates to construct μPADs. Wax printing [18], screen printing [19], laser cutting [20], photolithography [21], plasma treatment [22], and chemical vapor-phase deposition [23] are commonly used techniques for μPADs fabrication. In spite of providing fast fabrication and high resolution of hydrophobic hindrance and microfluidic direction, their routine application is limited by the need for expensive instruments and trained personnel. To solve such problems, some groups have developed simple techniques to construct μPADs. Cardoso et al. developed microfluidic devices by stamping paraffin barriers in paper platform for the colorimetric determination of nitrite [24]. Despite the simplicity of the technique, stamping is not without drawbacks, e.g., the high cost to generate a new metal stamp for each new prototype design. Pena-Pereira et al. used a commercially available permanent marker to plot the required hydrophobic barriers on the filter paper for μPADs fabrication [25]. This technique needs to plot the detection areas on both sides of the filter paper, and it requires careful alignment of the patterned stencils. Nurak et al. fabricated paper-based devices based on a lacquer spraying method for the determination of nickel (II) ion in waste water [26]. However, the disadvantage that is associated with the use of acrylic lacquer is the lack of flexibility of the resulting μPADs, due to the relatively fragile hydrophobic barriers.

Adhesive tape is a low cost consumable, which is often used in the fabrication of microfluidic devices. Martinez et al. fabricated three-dimensional (3D) μPADs by stacking alternating layers of paper and water-impermeable double-sided adhesive tape [27]. The layers of water-impermeable double sided tape separated channels in neighboring layers of paper, and holes cut into the tape allowed fluids to vertically flow. Yu et al. developed the 3D μPADs by stacking the paper layer between two layers of water-impermeable single-sided adhesive tapes [28]. The layers of tapes could eliminate the evaporation of the sample solution and decrease the influence of the external interference, such as wet air and dust. Mentele et al. fabricated the two-dimensional (2D) μPADs and covered one side of the μPADs with clear packing tape to prevent solution from leaking out underneath the paper during the assay [29]. Ren et al. developed a new double-layered acrylic microfluidic device based on a reversible tape-based mechanism [30]. In their work, hydrophobic patterns were formed in the adhesive tape under the help of a plastic mask and the microfluidic channels were formed inside the acrylic board while using a professional laser cutter. They use the adhesive tape as a separate layer for sealing or fluid flow controlling to fabricate multi-layered microfluidics. No work has been reported on utilizing the deposition of cut adhesive tape into the filter paper to fabricate single-layered paper microfluidics, which is a mask-free fabrication process without the usage of any expensive instruments (laser cutter, cutting plotter, screen printer, wax printer, 3D printer, etc.).

Nitrite is an important intermediate in biological systems and it can be used as food additives [31]. However, a remarkable degree of excess nitrite intake can cause a serious health hazard to the public, such as methemoglobinemia, occasional intoxications, and potential cancer [32]. What is more, nitrites are important in the diagnosis and detection of urinary system diseases, because they result from microorganisms that reduce nitrate to nitrous acid in urine, something that especially occurs in the urinary tract infection (UTI) [33]. Glucose is a vital resource for the human body to produce energy molecule Adenosine triphosphate (ATP), while high levels of glucose in human physiological fluids could lead to a number of metabolic disorders (e.g., diabetes mellitus) [34]. Although glucose monitoring in blood is common, urine glucose monitoring is also equally important, as it allows for us to monitor kidney function. In the condition, called renal glycosuria, the urine glucose levels might be higher, even if the blood glucose levels are at normal [35]. Therefore, nitrite and glucose were used as the analytes to demonstrate the assay performance of the developed μPADs.

In this paper, a simple, low-cost and highly-adaptable prototyping process for the fabrication of single-layered µPADs has been developed. The patterned double sided adhesive tape was dissolved by toluene and deposited into the filter paper at the same time. Different hydrophilic channels were obtained by changing the double sided adhesive tape with different design. The performance of the developed μPADs was demonstrated by individually performing colorimetric assays for nitrite and glucose. The detection limits for nitrite and glucose assays were 0.018 mM and 0.023 mM, respectively. In the multiplexed analysis of both analytes, the respective detection limits of 0.051 mM and 0.024 mM for nitrite and for glucose were achieved. Furthermore, recovery experiments were carried out by analyzing the concentrations of nitrite and glucose in artificial urine samples.

## 2. Experimental

### 2.1. Materials and Instruments

Whatman filter paper No. 1 was purchased from Whatman International Ltd. (Maidstone, England) and then used for μPADs fabrication. Water-impermeable double-sided adhesive tapes (brand Youbisheng, width = 24 mm and width = 50 mm) were bought from Shengneng Packaging Co., Ltd. (Hangzhou, China). The tapes are composed of a double-sided pressure sensitive adhesive layer and a protective film on one surface. The main composition of the pressure sensitive adhesive is styrene butadiene styrene block polymer and the thickness of the pressure sensitive adhesive layer is 75 μm. Craft punch (medium size), which is usually used for DIY Art & Craft Embossing, was purchased from Kamei Co., Ltd. (China) and it was used for cutting the desired microfluidic pattern into the adhesive tape. Glucose, sodium nitrite, sulfanilamide, N-(1-naphthyl) ethylenediamine dihydrochloride (NED), citric acid, potassium phosphate monobasic, potassium phosphate dibasic, NaNO_3_, Na_2_C_2_O_4_, Na_2_SO_4_, NaBr, NaHCO_3_, CH_3_COONa, Na_2_S_2_O_3_, NaF, Na_2_CO_3_, D(+)fructose, uric acid, urea, creatinine, KCl, NH_4_Cl, NaCl, and CaCl_2_·2H_2_O were of analytical grade and bought from Sinopharm Chemical Reagent Co., Ltd. (China). 3,3’,5,5’-tetramethylbenzidine (TMB), chitosan, glucose oxidase from Aspergillus niger (180 U/mg) were bought from Aladdin-Reagent Company (Shanghai, China). Peroxidase (from horseradish, 200 U/mg) was purchased from Macklin Biochemical Co. Ltd (Shanghai, China). Distilled water was used throughout. The standard stock solution of nitrite or glucose was prepared by dissolving sodium nitrite or glucose in distilled water and serially diluted for various concentrations. The nitrite assay is based on a colorimetric reaction while using Griess reagent [36]. The mixture of citric acid (330 mM), sulfanilamide (50 mM), and NED (10 mM) was prepared and used as the Griess reagent for nitrite detection. For glucose assay, the enzyme solution was freshly prepared containing glucose oxidase (GOx, 120 U/mL) and horseradish peroxidase (HRP, 30 U/mL) in 100 mM PBS (pH 6.0) for each experiment [37]. TMB solution (15 mM) was made in 95/5% (*v*/*v*) ethanol/water solvent. The chitosan-modified µPADs was used for nitrite and glucose detection, since the versatility of chitosan as an immobilization support has been successfully demonstrated by different authors for sensing studies in microfluidic scale [38,39,40]. The digital photos of the developed assays were taken by a smartphone (Redmi Note 4X, China) in a professional photography box (40 × 40 × 40 cm), including 84 brightness LED light beads inside (Shijiazhuang Ruying Film and Television Equipment Sales Co., Ltd., China). The cell phone, 8 cm away from the developed microfluidic device, was put on the top of the photography box for photography. The ImageJ software analyzed the average color values in RGB (Red, Green, and Blue) (National Institute of Health, USA). For the recovery test, artificial urine (pH 6.0) was used as the complex sample and prepared according to the reference [41], containing 1.103 g/L CaCl_2_·2H_2_O, 2.925 g/L NaCl, 2.25 g/L Na_2_SO_4_, 1.40 g/L KH_2_PO_4_, 1.60 g/L KCl, 1.00 g/L NH_4_Cl, 25 g/L urea, and 1.10 g/L creatinine.

### 2.2. The Fabrication of Single-Layered μPADs

**Figure 1:**
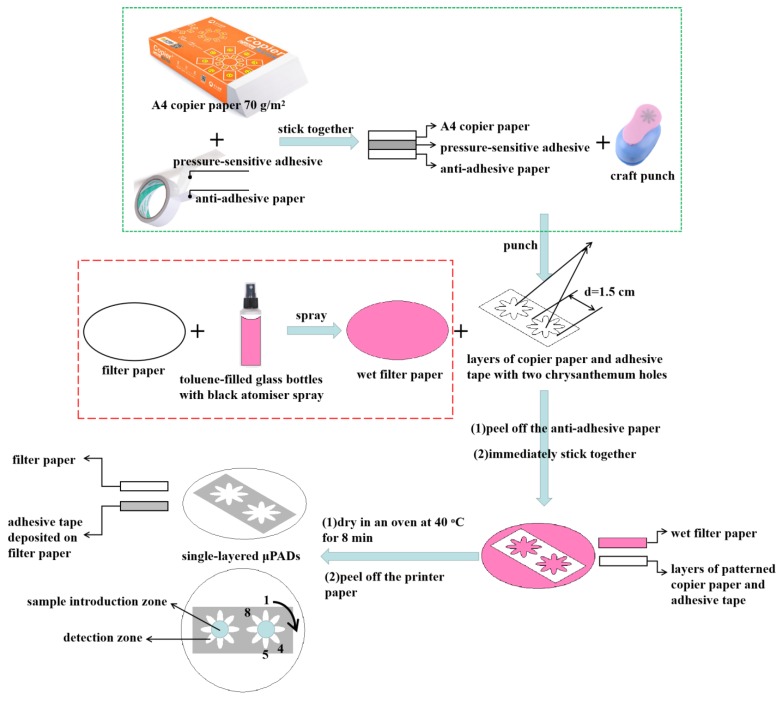
Schematic illustration of the fabrication process of single-layered single-layered paper-based microfluidic devices (μPADs).

Figure 1 shows the fabrication process of single-layered μPADs. The copier paper is covered by double-sided adhesive tape at the bottom and then was cut by a craft punch with the chrysanthemum design. The width of the chrysanthemum design was 1.5 cm. The filter paper was sprayed with toluene while using a glass spray bottle and then immediately stuck with the layers of copier paper and adhesive tape with two chrysanthemum holes. After that, the whole device was dried in an oven at 40 °C for 8 min. During the drying process, the adhesive tape was dissolved by toluene and then penetrated into the filter paper to form hydrophobic barriers. As a result, the adhesive tape was dissolved out and the copier paper was easily peeled off. The obtained filter paper with the deposition of patterned adhesive tape was used as the single-layered microfluidic device. As indicated in Figure 1, the white area, including the chrysanthemum flower shape, is hydrophilic in single-layered µPADs, while the grey area is hydrophobic. The center of the chrysanthemum flower was used as the sample introduction zone and the petals of the chrysanthemum were used as the detection zones. Using the craft punch with different designs can easily modify the hydrophilic channels’ pattern of µPADs.

### 2.3. Nitrite Detection

Initially, a solution of chitosan (0.5% *m*/*v*) was prepared in acetic acid 2% (*v*/*v*) and then kept stirring for 30 min. [37]. Subsequently, 0.6 μL of chitosan solution (0.5%, *w*/*v*) was added to each detection zone and then allowed to dry in an oven at 25 °C for 10 min. 0.6 μL of Griess reagent was then added to each detection zone and allowed to dry in an oven at 25 °C for 10 min. Finally, 12 μL of nitrite standard was added to the sample introduction zone. The nitrite in the standard solutions reacted with the Griess reagent for 10 min, and a purplish red coloured complex was formed in the detection zones. The images of the μPADs were acquired in JPEG format while using a smart phone and were then analyzed using the ImageJ software by selecting the whole detection zone and calculating the average color intensity.

### 2.4. Glucose Detection

At first, 0.6 μL of chitosan solution (0.5%, *w*/*v*) was added to each detection zone and allowed to dry in an oven at 25 °C for 10 min. After that, 0.6 μL of enzyme solution was dropped in each detection zone and then allowed to dry at 25 °C for 15 min. 0.6 μL of TMB solution was then dropped in the detection zone and allowed to dry at 25 °C for 15 min. 12 μL of glucose standard was added to the sample introduction zone. The glucose in the standard solutions reacted with the reagents for 10 min and a blue coloured complex was formed in the detection zones. The images of the μPADs were obtained by a smart phone and then were analyzed, as mentioned in the previous subsection.

### 2.5. Multiplexed Analysis of Nitrite and Glucose

Multiplexed analysis of nitrite and glucose was also investigated. Zones labelled 1–4 in Figure 1 were used for nitrite detection and zones 5–8 for glucose detection. For multiplexed analysis, 12 μL of the standard containing both nitrite and glucose was deposited in the sample introduction zone. The rest experimental procedures were the same as those mentioned previously.

### 2.6. Recovery Test

At first, 0.6 μL of chitosan solution (0.5%, *w*/*v*) was added to each detection zone and then dried in an oven at 25 °C for 10 min. After that, 0.6 μL of enzyme solution was dropped in the detection zones (1–4) and allowed to dry at 25 °C for 15 min. 0.6 μL of TMB solution and 0.6 μL of Griess reagent were then added to the detection zones (1–4) and zones (5–8), respectively, which were allowed to dry at 25 °C for 15 min. 12 μL of the artificial urine sample spiked with the standard solution containing both nitrite and glucose was added to the sample introduction zone, which produces a quick and even distribution of the sample by the capillary action towards the detection zones. The artificial urine sample reacted with the reagents for 10 min. and coloured complexes were formed in the detection zones. The images of the μPADs were obtained by a smart phone and then analyzed as mentioned in the previous subsection.

## 3. Results and Discussion

### 3.1. The Optimization for Fabrication Process

**Figure 2:**
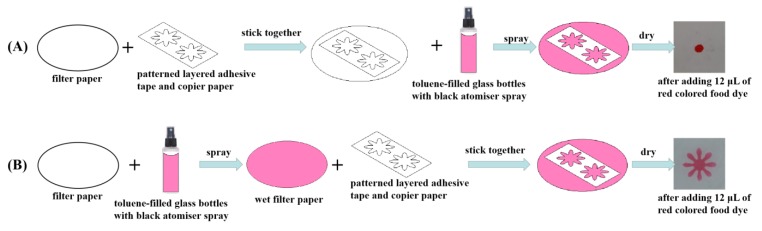
The optimization of the order in which toluene was sprayed. (**A**) The step of sticking together was put in the first order. (**B**) The step pf spraying toluene was put in the first order.

In order to obtain a clear hydrophobic boundary, the ordering in which toluene was sprayed was investigated for two scenarios: (1) spraying toluene on the filter paper after its sticking to the patterned copier paper; and, (2) spraying toluene on the filter paper before sticking. For the optimization experiment, the chrysanthemum flower design was chosen for the μPADs fabrication. Once the μPADs device is developed, 12 μL of red colored dye was pipetted into the sample introduction zone to show whether the desired hydrophilic regions can be obtained. As shown in Figure 2A, for the first scenario, the desired hydrophilic chrysanthemum area was not successfully constructed. The results demonstrated that toluene could dissolve the adhesive tape very well and the cellulose fibers in the filter paper allows for the dissolved adhesive tape by toluene to vertically and horizontally wick in the filter paper. As a result, it fails to produce a hydrophilic chrysanthemum flower on the filter paper. For the second scenario, as shown in Figure 2B, the hydrophilic chrysanthemum flower was achieved and the microfluidic device was successfully fabricated. The possible reason is that, in the second scenario, the flow of toluene reaches a temporary state of equilibrium on the filter paper before its adhesion to the layers of patterned copier paper and adhesive tape. Therefore, the adhesive tape might be vertically dissolved into the filter paper and rarely spread horizontally after their combination.

### 3.2. The Fabrication of μPADs

**Figure 3:**
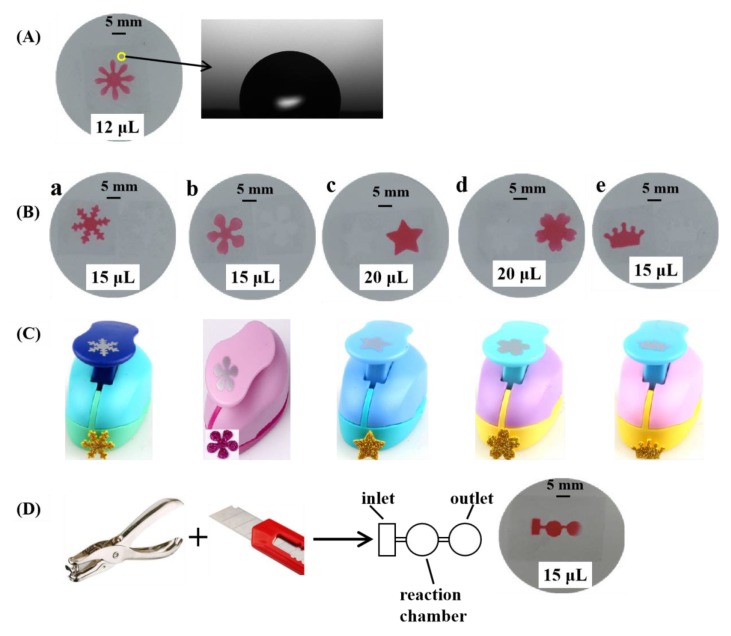
(**A**) Photograph of water droplets on the surface of prepared μPADs with the chrysanthemum design. (**B**) Single-layered microfluidic chip with simple designs. (**C**) The craft punch with different shapes: snowflake, flowers with five petals, pentagram, cherry blossom, and crown. (**D**) Single-layered μPAD with a complicated design after adding 15 μL of red colored food dye.

The wettability behavior of the prepared μPADs with the chrysanthemum design was investigated. Figure 3A shows the photograph of water droplets on the surface of the μPADs and the contact angle with water was 110.97 ± 3.62°. The filter paper originally had a hydrophilic property. After the adhesive tape was dissolved by toluene and penetrated into the filter paper, the surface of the filter paper became hydrophobic, showing a simple way to construct hydrophobic barriers on the filter paper. As we known, the ability to define accurate areas of hydrophobicity and hydrophilicity to control fluid flow is critically important in the successful fabrication of μPADs. The effectiveness of the new proposed method to form hydrophobic barriers is examined here for μPADs with simple designs, as shown in Figure 3B, by changing the craft punch with different shapes: snowflake, flowers with five petals, pentagram, cherry blossom, and crown (Figure 3C). For this purpose, 15 μL, 15 μL, 20 μL, 20 μL, and 15 μL of red colored food dye are pipetted into the sample introduction zone of the developed single-layered μPADs with simple designs, as shown in Figure 3B from a to e, respectively. The results indicate that the boundary between the hydrophobic and hydrophilic zones is well defined. The manufacturing cost of a single µPAD device with two chrysanthemum flowers (including materials) is ~US $0.051, indicating that the new technique is cost-efficient. The simple prototyping process developed in this work also fabricated a single-layered μPAD with a complicated design. The adhesive tape was cut by a hole punch and a knife. After that, the cut tape filled the filter paper and a single-layered μPAD was formed in the filter paper. The μPAD includes one inlet (3 mm × 6 mm), one reaction chamber (diameter 6 mm), and one outlet (diameter 6 mm). The width of the microchannels was found to be 774 µm, which indicated that the simple prototyping process could be used to fabricate μPADs with limited resources or where the access to standard fabrication technologies is restricted.

### 3.3. The Optimization of Sample Volume

**Figure 4:**
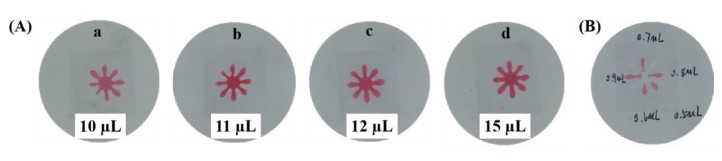
The optimization of the volume added in the central zone (**A**) and the detection zone (**B**).

The optimal volume added in the central zone and the detection zone was investigated and the results are shown in Figure 4. As shown in Figure 4A, the volume of the red food dye added in the central zone ranges from 10 to 15 µL. The complete chrysanthemum shape could not be observed in μPADs when 10 or 11 µL of red colored food dye was used. The whole chrysanthemum shape start to show up when 12 µL of red colored food dye was used. Thus, the optimal volume for sample introduction is taken to be 12 µL. Figure 4B shows the volume optimization results for the detection zone (petals of the chrysanthemum) by changing the volume from 0.5 to 0.9 µL. As indicated in the figure, the optimal volume for the detection zone is chosen to be 0.6 µL, which is the minimal value to show up one complete petal of the chrysanthemum.

### 3.4. Nitrite Assay Performance

**Figure 5:**
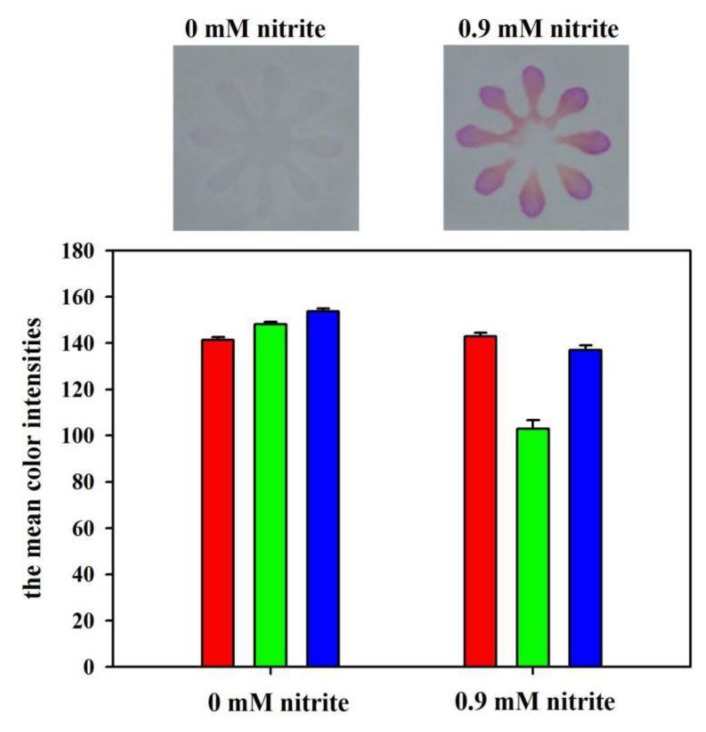
The color values for the developed μPADs after reacting with the blank and the nitrite standard (0.9 mM). The red, green, and blue vertical bars in the figure represent the mean colour intensities for the Red, Green, and Blue channels, respectively.

The feasibility of the developed μPADs for nitrite assay was demonstrated by measuring the color intensities in the RGB channels. The blank (0 mM) and the standard nitrite solution (0.9 mM) were used for the feasibility experiment. Figure 5 shows the color values in different color channels. It is noted that the mean color intensities for Red, Green, and Blue channels were 141, 148, and 154 for the blank, and 143, 103, and 137 for the 0.9 mM nitrite standard, respectively. Therefore, the absolute changes of the color intensity for Red, Green, and Blue channels for the two solutions were 2, 45, and 17, respectively. These results indicate that the Green channel intensity has the highest sensitivity for nitrite detection. Thus, Green channel intensity was chosen for nitrite analysis throughout the paper.

**Figure 6:**
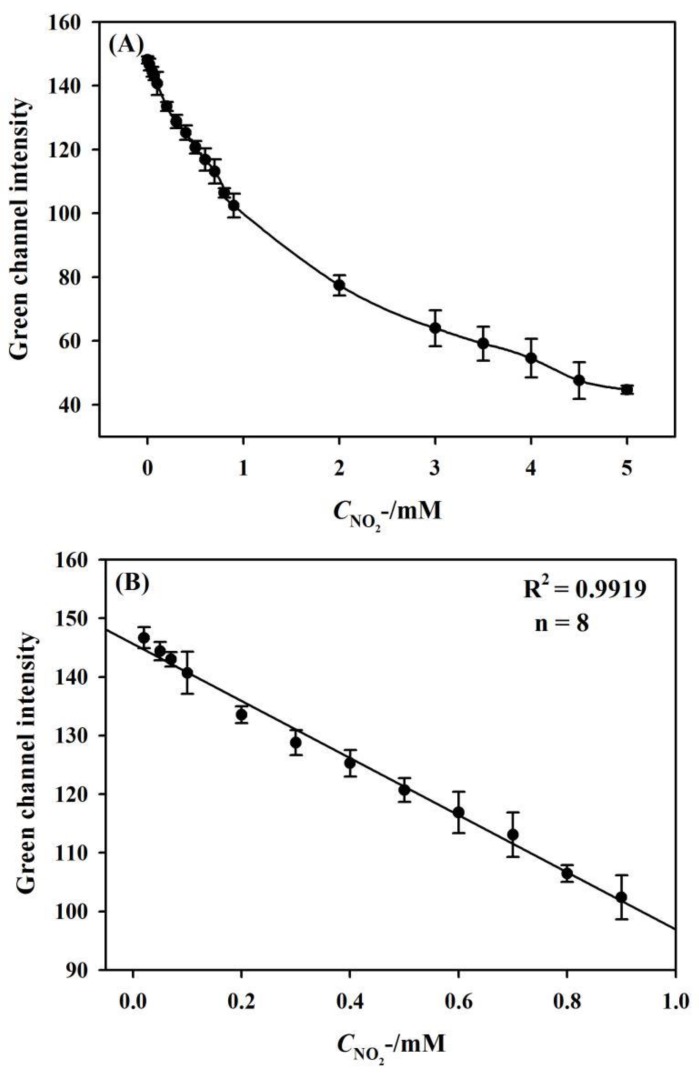
(**A**) Dependence of Green channel intensity on nitrite concentration. The concentrations are 0, 0.02, 0.05, 0.07, 0.1, 0.2, 0.3, 0.4, 0.5, 0.6, 0.7, 0.8, 0.9, 2.0, 3.0, 3.5, 4.0, 4.5, and 5.0 mM. (**B**) Calibrated range for nitrite detection.

To investigate the assay performance of the developed μPADs, a series of nitrite standards were spotted on the sample introduction zones and the digital photos of the devices were taken by a smart phone. It is noted from Figure 6A that the Green channel intensity decreases with the increase of nitrite concentration from 0 to 5 mM. A calibration curve between nitrite concentrations and corresponding color values exhibits an excellent linear range from 0.02 mM to 0.9 mM with R^2^ = 0.9919, as shown in Figure 6B. As per the IUPAC guidelines [42,43,44,45], the lower and upper limits of the limit of detection (LOD) interval (LOD_min_ and LOD_max_, respectively) correspond to the calibration samples with the lowest and largest extrapolated leverages to zero analyte concentration. For nitrite assay, a detection limit of 0.015 ± 0.004 mM is evaluated. 0.011 < x < 0.019 mM is the interval for LOD as LOD_min_ < x < LOD_max_. Table 1 provides a comparison of different sensors for the detection of nitrite. Although some work listed in Table 1 reported a low LOD, they require the usage of instruments (such as electrochemical workstation and UV-vis-NIR spectrophotometer) [46,47] make use of nanomaterials [47,48] or need longer response time [47,48]. The smartphone based sensor that was developed in this work achieved a comparable LOD for nitrite assay without using instruments and nanomaterials when compared to the previously reported sensors, which require a small sample volume (1.5 μL per detection zone). The response time (10 min.) is also comparable with most of the work reported in literature. Nilghaz et al. [49] developed a microfluidic cloth-based analytical device for nitrite detection with a shorter response time (2 min.). The possible reason is that cotton fabric shows better mixing uniformity between reagents and analyte across the detection zones. Our nitrite measurement range is 0.02~0.9 mM, or part of the wider range done by other groups [46,47,50,51]. However, our small measurement range is quite enough to detect bacteriuria that upon its occurrence would normally be indicated by nitrite level as low as 20 μM [52], which indicates good assay performance for nitrite detection.

### 3.5. Glucose Assay Performance

**Figure 7:**
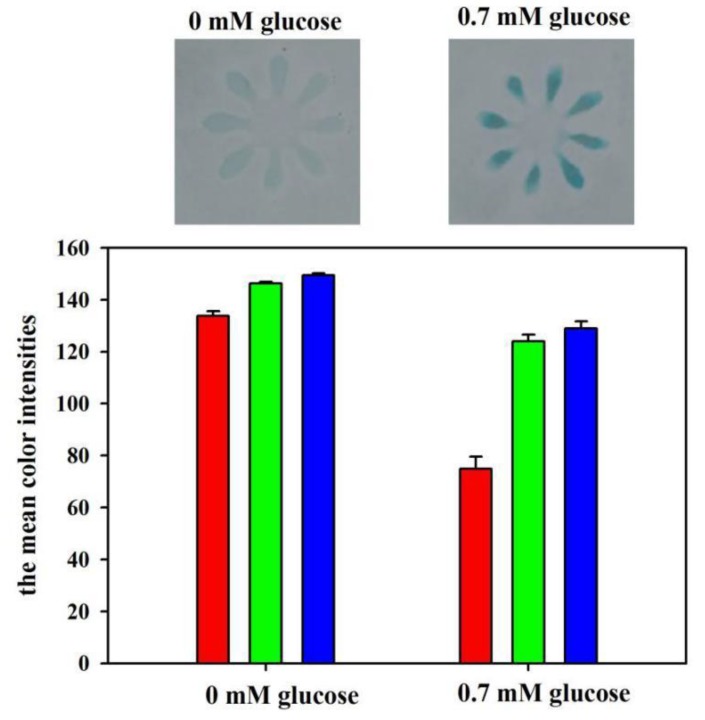
The color values for the developed μPADs after reacting with the blank and the glucose standard (0.7 mM). The red, green, and blue vertical bars in the figure represent the mean colour intensities for the Red, Green, and Blue channels, respectively.

The feasibility of the developed μPADs for glucose assay was also investigated. The blank (0 mM) and the standard glucose solution (0.7 mM) were used for the feasibility experiment. Figure 7 shows the color values in different channels. It is noted that the mean color intensities for Red, Green, and Blue channels were 134, 146, and 149 for the blank, and 75, 124, and 129 for the 0.7 mM glucose standard, respectively. The absolute changes of the color intensity for Red, Green, and Blue channels for the two solutions were 59, 22, and 20, respectively. These results indicate that the Red channel intensity has the highest sensitivity for glucose detection. Thus, Red channel intensity was chosen for glucose analysis throughout the paper.

**Figure 8:**
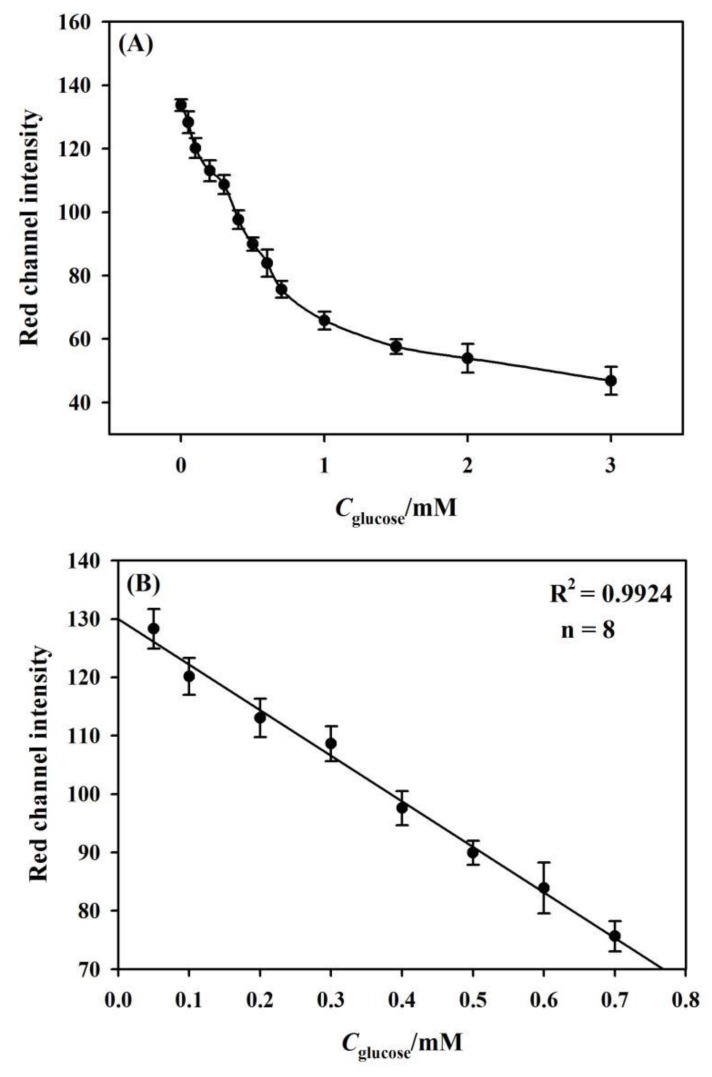
(**A**) Dependence of Red channel intensity on nitrite concentration. The concentrations are 0, 0.05, 0.1, 0.2, 0.3, 0.4, 0.5, 0.6, 0.7, 1.0, 1.5, 2.0, and 3.0 mM. (**B**) The linear fit plot of Red channel intensity and glucose from 0.05 mM to 0.7 mM.

The assay performance of the developed μPADs towards glucose assay was then investigated. It can be observed from Figure 8A that the Red channel intensity decreases with the increase of the glucose concentration that ranged from 0 mM to 3 mM. A calibration curve between glucose concentrations and corresponding color values exhibits an excellent linear range from 0.05 mM to 0.7 mM with R^2^ = 0.9924, as shown in Figure 8B. As per the IUPAC guidelines [42], a detection limit of 0.022 ± 0.006 mM is evaluated for glucose assay. 0.016 < x < 0.028 mM is the interval for LOD as LOD_min_ < x < LOD_max_. The parameters that were achieved in this work were compared to the data described in literature, who monitored glucose through enzymatic reactions with specific indicators, as demonstrated in Table 2. Although the work that was done by Gabriel et al. [37] reported a low LOD, both the longer response time and the larger sample volume are required. The LOD value obtained in this work is comparable with those reported in the literature. Furthermore, the response time (10 min.) and the sample volume (12 μL or 1.5 μL per detection zone) are also comparable with those reported in literature. Our glucose measurement range is 0.05~0.7 mM, or part of the wider range done by other groups that are listed in Table 2. However, our small measurement range is within the clinically relevant range for glucose assay in urine [52], indicating a good assay performance for glucose detection.

### 3.6. Multiplexed Analysis of Nitrite and Glucose

**Figure 9:**
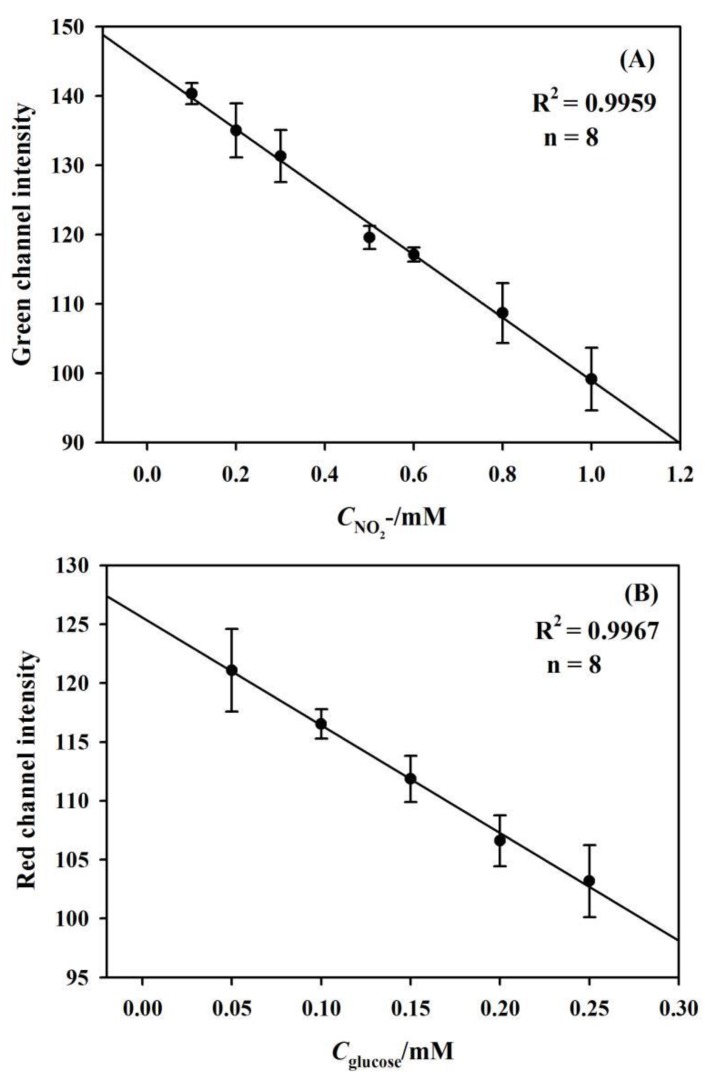
(**A**) The calibration plot of Green channel intensity and nitrite from 0.1 mM to 1.0 mM. (**B**) The calibration plot of Red channel intensity and glucose from 0.05 mM to 0.25 mM.

The multiplexed assay performance of the fabricated μPADs was investigated by depositing the standard solution containing both nitrite and glucose into the sample introduction zone. For the multiplexed assay, the eight detection zones (numbering 1–8 with 1–4 for nitrite detection and 5–8 for glucose detection) that were connected to a central sample deposition spot were created, as shown in Figure 1. Figure 9A shows the linear fit plot of the Green channel color value versus the concentration of nitrite that ranged from 0.1 to 1.0 mM (R^2^ = 0.9959). Figure 9B indicates that the sensing system exhibited a linear relationship between the Red channel color value and the glucose concentration in the range 0.05–0.25 mM (R^2^ = 0.9967). As per IUPAC guidelines [42], the detection limits of 0.048 ± 0.005 mM and 0.025 ± 0.006 mM are evaluated for nitrite and glucose, respectively. These results verify the success of the multiplexed assay in this novel microfluidic format.

### 3.7. Specificity, Reproducibility and Stability

**Figure 10:**
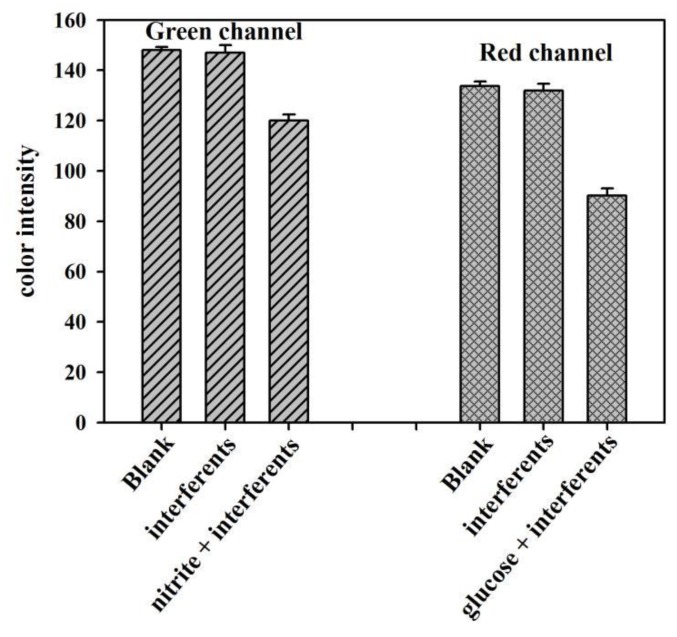
Specificity of the developed μPADs. The concentration of nitrite or glucose is 0.5 mM and the concentrations of the other interferents are all 50 mM.

To check the potential interferences in urine that can affect the analytical signal of the μPADs, we specifically studied the effect of some species common in the samples. NO_3_^−^, C_2_O_4_^2−^, SO_4_^2−^, Br^−^, HCO_3_^−^, CH_3_COO^−^, S_2_O_3_^2−^, F^−^, H_2_PO_4_^−^, CO_3_^2−^, D(+)fructose, uric acid, urea, NaCl, and CaCl_2_ were chosen as the interferents [48,51,61]. Comparing the color intensity between the solutions containing interferents only, and the mixture of nitrite or glucose with other interferents investigated the specificity of the developed μPADs. As shown in Figure 10, the response signals caused by the interferents are almost the same as that of blank sample, whereas the mixture of nitrite or glucose with other interferents gives an obvious change in the color intensity. These results suggest that the developed sensor has excellent specificity for nitrite and glucose assays.

**Figure 11:**
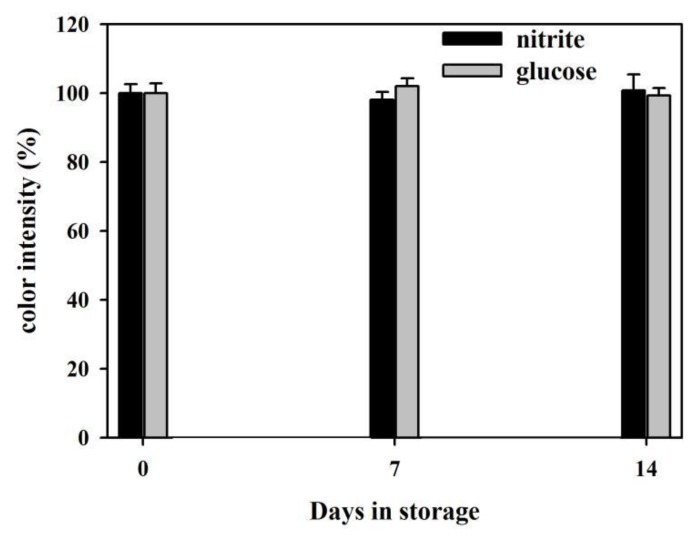
Storage stability of the developed μPADs with immobilized reagents in the refrigerator (shown as % change with 100% being the response at day 0).

The reproducibility of the μPADs was evaluated by the measures of the coefficient of variation (CV) for intra-assay and inter-assay [43]. The CV is a dimensionless number that is defined as the standard deviation of a set of measurements divided by the mean of the set. The μPADs that were fabricated on the same day were recognized as the same batch, while the μPADs fabricated on different dates were recognized as different batches. The μPAD with one chrysanthemum flower was recognized as one sensor. For intra-assay, six standards, including the nitrite standards (0.1 mM, 0.5 mM, 0.9 mM) and the glucose standards (0.1 mM, 0.5 mM, 0.7 mM), were labeled as solution# 1, 2, 3, 4, 5, and 6, each of which was tested in the detection zone 1 of three sensors in the same batch. Data measured for each solution were recorded and they are summarized in Table 3. The intra-assay CVs for nitrite and glucose for n = 3 were 2.93% and 3.28%, respectively, which are less than 10%, and thus reflect good reproducibility of the results. For inter-assay, the standards with high, medium, and low concentrations of the analyte were tested in the detection zone 1 of the sensors, which are run in triplicate on three different batches to monitor batch-to-batch variation. The color intensity in the detection zone 1 of every sensor was measured and recorded in Table 4. The mean color intensities for high, medium and low concentrations of analyte are calculated and then used to calculate the mean of means and std. of means. The CVs for high, medium, and low concentrations of analyte are calculated by the std. of means divided by the mean of means. The average of the CVs for high, medium, and low concentrations of analyte is reported as the inter-assay CV. The inter-assay CVs for n = 3 are 4.18% ((4.37 + 4.91 + 3.26)/3) and 4.63% ((5.49 + 4.23 + 4.18)/3) for nitrite and glucose, respectively, which indicates the good batch-to-batch reproducibility. The storage stability of the developed μPADs in which the reagents were immobilized on the detection zones was evaluated by measuring the colorimetric response. The solutions of 0.5 mM nitrite and 0.5 mM glucose were used in these tests. Figure 11 shows that the μPADs remain active after a two-week storage in the refrigerator (4 °C) and the color intensities have no significant changes. These demonstrate that the developed μPADs have satisfactory storage stability.

### 3.8. Recovery Test

The applicability and reliability of the developed μPADs were evaluated through recovery experiments. For this purpose, the artificial urine samples were spiked with standard solutions that contained both nitrite and glucose and the results are listed in Table 5. The nitrite recoveries range from 101% to 112%, which are greater than 100%. The possible reason for the high nitrite recoveries is that the substances in urine lead to a change in pH, which is beneficial for the Griess reaction [62]. The glucose recoveries range from 91% to 108%. The low recoveries of glucose might be caused by the reduction of H_2_O_2_ by the reducing substances in urine, which decreased the amount of H_2_O_2_ to react with TMB. In addition, the %RSD of the recovery results is below 5%. These satisfactory results show that the assay is reliable and it indicates great promise for the detection of nitrite and glucose in real samples.

## 4. Conclusions

In this paper, we developed a new method for fabricating single-layered µPADs. Within this scheme, the cut adhesive tape was dissolved by toluene and penetrated into the filter paper to form hydrophobic barriers, defining accurate areas of hydrophobicity and hydrophilicity. This makes the fabrication process simple and efficient. During the entire manufacturing process for µPADs, no expensive instruments (laser cutter, screen printer, wax printer, etc.) are used and the cost of materials is about US $0.051 per microfluidic chip. The performance of the developed μPADs was demonstrated by implementing colorimetric assays for nitrite and glucose. The detection limits for individual assays were 0.015 ± 0.004 mM and 0.022 ± 0.006 mM for nitrite and glucose, respectively. For multiplexed assays, the detection limits were 0.048 ± 0.005 mM and 0.025 ± 0.006 mM for nitrite and glucose, respectively. In addition, the device had satisfactory recoveries in artificial urine samples, providing a highly useful and practical platform for the monitoring of nitrite and glucose in real samples. In spite of the advantages of the developed µPADs (i.e., high portability, applicability, and cost-effectiveness), the massive production of µPADs with high resolution still needs to resort to the usage of some automatic cutting machines. In addition, more environmental friendly solvents will be tried for the µPADs fabrication in the future work.

## Figures and Tables

**Table 1:** Comparison of the parameters achieved for nitrite assay performed on sensors found in literature.

Sensing Reagents/Materials	Method	Response Time (min)	Volume (μL)	Linear Range (mM)	LOD (mM)	Refs
Copper nanoparticles modified electrodes	Electrochemistry	0.05		0.05~30	0.02	[46]
Functionalized gold nanoparticles	colorimetric	30		0~0.11	0.022	[47]
Griess reagent	colorimetric	2	15		0.16	[49]
Griess reagent	colorimetric				0.087	[53]
Griess reagent	Colorimetric	2	1.40	0.156~1.25		[50]
Iridium(III) complexes	Colorimetric	240		0.05~20	0.05	[51]
Gold nanoparticle	Colorimetric	25	100		0.022	[48]
Griess reagent	Colorimetric	20			0.06	[54]
Griess reagent	Colorimetric	10	12	0.02~0.9	0.015 ± 0.004	This work

**Table 2:** Comparison of the parameters achieved for glucose assay performed on sensors found in literature.

Sensing Reagents/Materials	Method	Response Time (min)	Sample Volume (μL)	Linear Range (mM)	LOD (mM)	Refs
GOx, HRP, 4-AAP/DHBS	colorimetric	15	70	0.1~1.0	0.023	[37]
GOx, HRP, TMB	colorimetric	15	70	1.0~5.0	0.057	[37]
GOx, HRP, TMB	colorimetric	15	5	0.1~1	0.05	[55]
GOx, HRP, 4-AAP/TOPS	colorimetric	30	2	0.3~1	0.213	[56]
GOx, HRP, 4-AAP/DHBS	colorimetric		40	2.0~12	0.7	[57]
GOx, HRP, 4-AAP/MAOS	colorimetric	1	15	0.3~8.0	0.3	[58]
GOx, HRP, KI/trehalose	colorimetric	30	10	0.5~10	0.5	[59]
GOx, Ceria nanoparticles	colorimetric	10		0.5~100	0.5	[60]
GOx, HRP, KI	colorimetric		10	0~27.8	1.4	[23]
GOx, HRP, TMB	Colorimetric	10	12	0.05~0.7	0.022 ± 0.006	This work

4-aminoantipyrine (AAP), 3,5-dichloro-2-hydroxy-benzenesulfonic acid (DHBS); N-ethyl-N(3-sulfopropyl)-3-methyl-aniline sodium salt (TOPS); N-ethyl-N-(2-hydroxy-3-sulfopropyl)-3,5-dimethylaniline sodium salt monohydrate (MAOS).

**Table 3:** Intra-assay coefficients of variation (CV).

**Solution #**	**Green Color Intensity**			**Mean Green Color Intensity**	**Std. Dev.**	**CV (%)**
1	136.26	144.34	140.54	140.38	4.04	2.88
2	121.63	117.63	122.09	120.45	2.45	2.03
3	105.51	103.44	97.86	102.27	3.96	3.87
**Solution #**	**Red Color Intensity**			**Mean Red Color Intensity**	**Std. Dev.**	**CV (%)**
4	116.10	122.91	121.27	120.09	3.55	2.96
5	88.80	86.32	92.35	89.16	3.03	3.40
6	78.66	73.88	74.35	75.63	2.63	3.48

**Table 4:** Inter-assay coefficients of variation (CV).

**Nitrite Conc. (mM)**	**Batch #/Sensor #/Green Color Intensity**			**Mean Green Color Intensity**	**Mean of Means**	**Std. of Means**	**CV (%)**
High (0.9)	1/1/105.51	1/2/103.44	1/3/97.86	Batch #1: 102.27	100.83	4.41	4.37
High (0.9)	2/1/98.22	2/2/96.14	2/3/93.28	Batch #2: 95.88			
High (0.9)	3/1/102.58	3/2/106.79	3/3/103.66	Batch #3: 104.34			
Medium (0.5)	1/1/116.63	1/2/115.60	1/3/122.09	Batch #1: 118.11	120.70	5.93	4.91
Medium (0.5)	2/1/129.35	2/2/130.21	2/3/122.89	Batch #2: 127.48			
Medium (0.5)	3/1/114.58	3/2/116.42	3/3/118.49	Batch #3: 116.50			
Low (0.1)	1/1/136.26	1/2/144.34	1/3/140.54	Batch #1: 140.38	141.12	4.60	3.26
Low (0.1)	2/1/145.36	2/2/146.30	2/3/142.11	Batch #2: 144.59			
Low (0.1)	3/1/130.58	3/2/139.41	3/3/136.22	Batch #3: 135.40			
**Glucose Conc. (mM)**	**Batch #/Sensor #/Red Color Intensity**			**Mean Red Color Intensity**	**Mean of Means**	**Std. of Means**	**CV (%)**
High (0.7)	1/1/78.66	1/2/73.88	1/3/74.35	Batch #1: 75.63	76.28	4.19	5.49
High (0.7)	2/1/80.25	2/2/79.48	2/3/82.55	Batch #2: 80.76			
High (0.7)	3/1/72.53	3/2/73.88	3/3/70.96	Batch #3: 72.46			
Medium (0.5)	1/1/88.80	1/2/86.32	1/3/92.35	Batch #1: 89.16	88.72	3.75	4.23
Medium (0.5)	2/1/85.69	2/2/82.11	2/3/86.52	Batch #2: 84.77			
Medium (0.5)	3/1/94.26	3/2/90.81	3/3/91.63	Batch #3: 92.23			
Low (0.1)	1/1/116.1	1/2/122.91	1/3/121.27	Batch #1: 120.09	119.74	5.00	4.18
Low (0.1)	2/1/126.32	2/2/122.20	2/3/125.16	Batch #2: 124.56			
Low (0.1)	3/1/112.56	3/2/114.79	3/3/116.36	Batch #3: 114.57			

**Table 5:** Recovery test in artificial urine samples.

nitrite	**Sample No.**	**Added (mM)**	**Found (mM)**	**RSD (%)**	**Recovery (%)**
	1	0.1	0.105	1.7	105
	2	0.5	0.548	3.4	110
	3	0.7	0.784	4.6	112
	4	0.9	0.906	1.8	101
glucose	**Sample No.**	**Added (mM)**	**Found (mM)**	**RSD (%)**	**Recovery (%)**
	1	0.05	0.0480	1.8	96
	2	0.1	0.0933	1.8	93
	3	0.15	0.137	3.0	91
	4	0.2	0.215	4.5	108
